# Supplementary material for: Emerging and Novel Viruses in Passerine Birds
Source: Microorganisms. 2023 Sep 20;11(9):2355. doi: 10.3390/microorganisms11092355 (PMC10536639; doi:10.3390/microorganisms11092355)
Supplement: Supplementary file 1 [file microorganisms-11-02355-s001.zip › Supplementary_Material_Table S1.pdf]

EMERGING AND NOVEL VIRUSES IN PASSERINE BIRDS

Richard AJ Williams[1,3]; Christian Sánchez[1], Ana Doménech [2,3], Ricardo Madrid[1,3]; Sergio Fandiño[2,3]; Pablo Cea-Calleja[1,3];, Esperanza Gomez-Lucia[2,3], Laura Benítez[1,3]

[1]Department of Genetics, Physiology, and Microbiology, School of Biology, Complutense University of Madrid (UCM), C. de José Antonio Nováis, 12, 28040, Madrid, Spain

[2]Deparment of Animal Health, Veterinary Faculty, Complutense University of Madrid, Av. Puerta de Hierro, s/n, 28040, Madrid, Spain

[3] "Animal viruses" Research Group, Complutense University of Madrid, Madrid, Spain

SUPPLEMENTARY MATERIAL: TABLES S1: List of bird species

Table S1: List of bird species, or more general bird names, cited in this review, following Clements 2021 [1]. Note: this list aims to provide background on the taxonomy of birds listed in this review.

Common species name	Scientific name	Avian family	Order
American cliff swallow	*Petrochelidon pyrrhonota*	Hirundidae	Passeriformes
American crow	*Corvus brachyrhynchos*	Corvidae	Passeriformes
American robin	*Turdus migratorius*	Turdidae	Passeriformes
Australian magpie	*Gymnorhina tibicen*	Artamidae	Passeriformes
Australian raven	*Corvus coronoides*	Corvidae	Passeriformes
Black-billed magpie	*Pica hudsonia*	Corvidae	Passeriformes
Black-capped chickadee	*Poecile atricapillus*	Paridae	Passeriformes
Black-faced quailfinch	*Ortygospiza atricollis*	Estrildidae	Passeriformes
Black-headed antbird	*Percnostola rufifrons*	Thamnophilidae	Passeriformes

Black-naped monarch	*Hypothymis azurea*	Monarchidae	Passeriformes
Black-rumped waxbill	*Estrilda troglodytes*	Estrildidae	Passeriformes
Black-throated finch	*Poephila cincta*	Estrildidae	Passeriformes
Blue jay	*Cyanocitta cristata*	Corvidae	Passeriformes
Brambling	*Fringilla montifringilla*	Fringillidae	Passeriformes
Cardinals and allies		Cardinalidae	Passeriformes
Chestnut-bellied seed-finch	*Sporophila angolensis*	Estrildidae	Passeriformes
Chinese bulbul	*Pycnonotus sinensis*	Pynonotidae	Passeriformes
Common blackbird	*Turdus merula*	Turdidae	Passeriformes
Common chaffinch	*Fringilla coelebs*	Fringillidae	Passeriformes
Cormorants		Sulidae	Suliformes
Crows, jays, and magpies		Corvidae	Passeriformes
Daurian redstart	*Phoenicurus auroreus*	Muscicapidae	Passeriformes
Domestic canary	*Serinus canaria* forma *domestica*	Fringillidae	Passeriformes
Domestic chicken or fowl	*Gallus gallus domesticus*	Phasianidae	Galliformes
Ducks, geese, swans (waterfowl)		Anatidae	Anseriformes
Dunnock	*Prunella modularis*	Prunellidae	Passeriformes
Eastern spinebill	*Acanthorhynchus tenuirostris*	Meliphagidae	Passeriformes
Waxbills and allies		Estrildidae	Passeriformes
Eurasian blackcap	*Sylvia atricapilla*	Sylviidae	Passeriformes
Eurasian blue tit	*Cyanistes caeruleus*	Paridae	Passeriformes
Eurasian hawfinch	*Coccothraustes coccothraustes*	Fringillidae	Passeriformes
Eurasian jackdaw	*Corvus monedula*	Corvidae	Passeriformes
Eurasian robin	*Erithacus rubecula*	Muscicapidae	Passeriformes
Eurasian starling		Sturnidae	Passeriformes
European goldfinch	*Carduelis carduelis*	Fringillidae	Passeriformes
European greenfinch	*Chloris chloris*	Fringillidae	Passeriformes
Falcons (raptors)		Falconidae	Falconiformes
Ferruginous-backed antibird	*Myrmoderus ferruginea*	Thamnophilidae	Passeriformes

Goshawk	*Accipiter gentilis*	Accipitridae	Accipitriformes
Gouldian finch	*Chloebia gouldiae*	Estrildidae	Passeriformes
Gray-backed thrush	*Turdus hortulorum*	Turdidae	Passeriformes
Great tit	*Parus major*	Paridae	Passeriformes
Green-and-gold tanager	*Tangara schrankii*	Thraupidae	Passeriformes
Grey pileated finch	*Coryphospingus pileatus*	Thraupidae	Passeriformes
Gulls		Laridae	Charadriiformes
Hawaiian 'elepaio	*Chasiempis sandwichensis*	Monarchidae	Passeriformes
Hawks, eagles and kites (raptors)		Accipitridae	Accipitriformes
Herons, egrets and bitterns		Ardeidae	Suliformes
Hooded crow	*Corvus cornix*	Corvidae	Passeriformes
House finch	*Haemorhous mexicanus*	Fringillidae	Passeriformes
House sparrow	*Passer domesticus*	Passeridae	Passeriformes
Icterids (New world blackbirds)		Icteridae	Passeriformes
Indian myna	*Acridotheres tristis*	Sturnidae	Passeriformes
Jacky winter	*Microeca fascinans*	Petroicidae	Passeriformes
Japanese white-eye	*Zosterops japonicus*	Zosteropidae	Passeriformes
Java sparrow	*Lonchura oryzivora*	Estrildidae	Passeriformes
Kurrichane Thrush	*Turdus libonyana*	Turdidae	Passeriformes
Larks		Alaudidae	Passeriformes
Little greenbul	*Eurillas virens*	Pynonotidae	Passeriformes
Long-tailed finch	*Poephila acuticauda*	Estrildidae	Passeriformes
Magpie lark	*Grallina cyanoleuca*	Monarchidae	Passeriformes
Marsh tit	*Poecile palustris*	Paridae	Passeriformes
Masked finch	*Poephila personata*	Estrildidae	Passeriformes
New Zealand robin	*Petroica australis*	Petroicidae	Passeriformes
Noisy miner	*Manorina melanocephala*	Meliphagidae	Passeriformes
Old world flycatchers		Muscicapidae	Passeriformes

Old world sparrows		Passeridae	Passeriformes
Orchard oriole	*Icterus spurius*	Icteridae	Passeriformes
Owls		Strigidae	Strigiformes
Pale thrush	*Turdus palidus*	Turdidae	Passeriformes
Parrots		Psittacidae	Psittaciformes
New world warblers	Parulidae	Parulidae	Passeriformes
Pigeons		Columbidae	Columbiformes
Weavers	Ploceidae	Ploceidae	Passeriformes
Quail (common name applied to species from two families in order Galliformes)		Phasianidae	Galliformes; Odontophoridae
Red avadavat	*Amandava amandava*	Estrildidae	Passeriformes
Red-billed fire finch	*Lagonosticta senegala*	Estrildidae	Passeriformes
Red-billed quelea	*Quelea quelea*	Ploceidae	Passeriformes
Red-throated parrot finch	*Erythrura psittacea*	Estrildidae	Passeriformes
Red-whiskered bulbul	*Pycnonotus jocosus*	Pynonotidae	Passeriformes
Reed bunting	*Emberiza schoeniclus*	Emberizidae	Passeriformes
Rock dove	*Columba livia*	Columbidae	Columbiformes
Rufous-collared sparrow	*Zonotrichia capensis*	Emberizidae	Passeriformes
Rufous-rumped foliage-gleaner	*Philydor erythrocercum*	Furnariidae	Passeriformes
Rufous-throated antbird	*Gymnopithys rufigula*	Thamnophilidae	Passeriformes
Short-toed lark	*Calandrella rufescens*	Alaudidae	Passeriformes
Siberian accentor	*Prunella montanella*	Prunellidae	Passeriformes
Silvereye	*Zosterops lateralis*	Zosteropidae	Passeriformes
Song thrush	*Turdus philomelos*	Turdidae	Passeriformes
Southern house wren	*Troglodytes musculus*	Troglodytidae	Passeriformes
Spotless starling		Sturnidae	Passeriformes
Starlings		Sturnidae	Passeriformes
Superb starling	*Lamprotornis superbus*	Sturnidae	Passeriformes

Swallows		Hirundidae	Passeriformes
Red-flanked bluetail	*Tarsiger cyanurus*	Muscicapidae	Passeriformes
Thrushes	*Turdus* sp.	Turdidae	Passeriformes
Tomtit	*Petroica macrocephala*	Petroicidae	Passeriformes
Tree sparrow	*Passer montanus*	Passeridae	Passeriformes
True finches		Fringillidae	Passeriformes
Turkey	*Meleagris gallopavo*	Phasianidae	Galliformes
Violet-eared waxbill	*Granatina granatina*	Estrildidae	Passeriformes
Vitelline masked weaver	*Ploceus vitellinus*	Ploceidae	Passeriformes
Waders and shorebirds		Charadriidae; Scolopaciidae (etc.)	Charadriiformes
Whinchat	*Saxicola rubetra*	Muscicapidae	Passeriformes
White-plumed antbird	*Pithys albifrons*	Thamnophilidae	Passeriformes
White-rumped munia	*Lonchura striata*	Estrildidae	Passeriformes
White-throated laughingthrush	*Pterorhinus albogularis*	Leiothrichidae	Passeriformes
Wood thrush	*Hylocichla mustelina*	Turdidae	Passeriformes
Yellow wagtail	*Motacilla flava*	Motacillidae	Passeriformes
Yellow-browed bunting	*Emberiza chrysophrys*	Emberizidae	Passeriformes
Yellow-browed warbler	*Phylloscopus inornatus*	Phylloscopidae	Passeriformes
Yellow-throated bunting	*Emberiza elegans*	Emberizidae	Passeriformes
Yellow-winged pytilia	*Pytilia hypogrammica*	Estrildidae	Passeriformes
Zebra finch	*Taeniopygia guttata*	Estrildidae	Passeriformes

1. Clements, J.; Schulenberg, T.; Iliff, M.; Billerman, S.; Fredericks, T.; Gerbracht, J.; Woods, C. Checklist of Birds of the World Available online: https://www.birds.cornell.edu/clementschecklist/download/. (accessed on 19 July 2023).
