# Supplementary material for: Emerging and Novel Viruses in Passerine Birds
Source: Microorganisms. 2023 Sep 20;11(9):2355. doi: 10.3390/microorganisms11092355 (PMC10536639; doi:10.3390/microorganisms11092355)
Supplement: Supplementary file 1 [file microorganisms-11-02355-s001.zip › Supplementary_Material_Table S2.pdf]

EMERGING AND NOVEL VIRUSES IN PASSERINE BIRDS

Richard AJ Williams[1,3]; Christian Sánchez[1], Ana Doménech [2,3], Ricardo Madrid[1,3]; Sergio Fandiño[2,3]; Pablo Cea-Calleja[1,3]; Esperanza Gomez-Lucia[2,3], Laura Benítez[1,3]

[1]Department of Genetics, Physiology, and Microbiology, School of Biology, Complutense University of Madrid (UCM), C. de José Antonio Nováis, 12, 28040, Madrid, Spain

[2]Deparment of Animal Health, Veterinary Faculty, Complutense University of Madrid, Av. Puerta de Hierro, s/n, 28040, Madrid, Spain

[3] "Animal viruses" Research Group, Complutense University of Madrid, Madrid, Spain

SUPPLEMENTARY MATERIAL: TABLES S2: List of avian families

Table S2: List of avian families mentioned in this review, following Clements 2021 [1], concentrating on families from order Passeriformes, but also including non-Passeriformes at the end of the table. The number of species belonging to the family is shown. Additional information is available for WNV and USUV (Table S3 and Table S4), and Poxviridae [2].

**Order**	**Latin avian family name**	**Common family name**	**Number of species in family**	**Viruses cited in this family in this review**
Passeriformes	Alaudidae	Larks	99	*Hepeviridae; Poxviridae*
Passeriformes	Artamidae	Woodswallows, Bellmagpies, and Allies	24	*Circoviridae*
Passeriformes	Cardinalidae	Cardinals and Allies	49	*Hepeviridae*
Passeriformes	Corvidae	Crows, Jays, and Magpies	128	*Bornaviridae; Circoviridae; Flaviviridae; Herpesviridae; Orthomyxoviridae; Paramyxoviridae*

Passeriformes	Emberizidae	Yellow-browed bunting	44	*Caliciviridae; Retroviridae; Rotaviridae; Paramyxoviridae*
Passeriformes	Estrildidae	Waxbills and allies	140	*Adenoviridae; Bornaviridae; Circoviridae; Coronaviridae; Orthomyxoviridae; Paramyxoviridae ; Picornaviridae; Rotaviridae; Togaviridae;*
Passeriformes	Fringillidae	True finches	229	*Adenoviridae; Astroviridae; Bornaviridae; Caliciviridae; Circoviridae; Flaviviridae; Hepeviridae; Herpesviridae; Papillomaviridae; Poxviridae; Rotaviridae*
Passeriformes	Furnariidae	Ovenbirds and Woodcreepers	306	*Anelloviridae*
Passeriformes	Hirundidae	Swallows	86	*Orthomyxoviridae; Togaviridae*
Passeriformes	Icteridae	Icterids (New world blackbirds)	105	*Orthomyxoviridae; Togaviridae*
Passeriformes	Leiothrichidae	Laughingthrushes and Allies	143	*Retroviridae*
Passeriformes	Meliphagidae	Honeyeaters	190	*Adenoviridae*
Passeriformes	Monarchidae	Monarch Flycatchers	100	*Astroviridae; Poxviridae; Togaviridae;*
Passeriformes	Motacillidae	Yellow wagtail	67	*Paramyxoviridae*
Passeriformes	Muscicapidae	Old world flycatchers	327	*Caliciviridae; Flaviviridae; Picornaviridae; Paramyxoviridae; Retroviridae*
Passeriformes	Paridae	Tits, Chickadees, and Titmice	63	*Adenoviridae; Herpesviridae; Poxviridae; Picornaviridae; Retroviridae*
Passeriformes	Parulidae	New world warblers	111	*Adenoviridae*

Passeriformes	Passeridae	Old world sparrows	43	*Adenoviridae; Coronaviridae; Flaviviridae; Orthomyxoviridae; Paramyxoviridae; Pneumoviridae; Poxviridae; Retroviridae; Togaviridae,*
Passeriformes	Petroicidae	Australasian Robins	49	*Caliciviridae; Circoviridae; Togaviridae*
Passeriformes	Phylloscopidae	Leaf Warblers	79	*Retroviridae*
Passeriformes	Ploceidae	Weavers and Allies	118	*Adenoviridae; Coronaviridae; Orthomyxoviridae; Paramyxoviridae*
Passeriformes	Prunellidae	Accentors	13	*Caliciviridae*
Passeriformes	Pycnonotidae	Bulbuls	151	*Coronaviridae; Flaviviridae*
Passeriformes	Sturnidae	Starlings	123	*Caliciviridae; Circoviridae; Coronaviridae; Herpesviridae; Orthomyxoviridae; Paramyxoviridae*
Passeriformes	Sylviidae	Sylviid Warblers, Parrotbills, and Allies	69	*Herpesviridae; Orthomyxoviridae*
Passeriformes	Thamnophilidae	Typical Antbirds	237	*Anelloviridae; Flaviviridae; Picornaviridae;* hepe-like viruses
Passeriformes	Thraupidae	Tanagers and Allies	381	*Parvoviridae; Retroviridae*
Passeriformes	Troglodytidae	Wrens	86	*Paramyxoviridae*
Passeriformes	Turdidae	Thrushes and Allies	174	*Caliciviridae; Coronaviridae; Flaviviridae; Herpesviridae; Orthomyxoviridae; Picornaviridae; Rotaviridae; Togaviridae;* hepe-like viruses
Passeriformes	Zosteropidae	White-eyes, Yuhinas, and Allies	142	*Coronaviridae;* hepe-like viruses
Accipitriformes	Accipitridae	Hawks, eagles and kites (raptors)	250	*Flaviviridae; Orthomyxoviridae*
Anseriformes	Anatidae	Ducks, geese, swans (waterfowl)	174	*Orthomyxoviridae; Parvoviridae; Togaviridae*

Charadriiformes	Scolopaciidae	Waders and shorebirds	97	*Orthomyxoviridae*
Charadriiformes	Laridae	Gulls and Terns	99	*Orthomyxoviridae*
Columbiformes	Columbidae	Pigeons	348	*Circoviridae; Herpesviridae; Paramyxoviridae;*
Falconiformes	Falconidae	Falcons (raptors)	65	*Orthomyxoviridae*
Galliformes	Phasianidae	Pheasants, Grouse, and Allies	181	*Anelloviridae; Flaviviridae; Poxviridae; Togaviridae*
Pelecaniformes	Ardeidae	Herons, egrets and bitterns	68	*Flaviviridae*
Psittaciformes	Psittacidae	Parrots	175	*Circoviridae; Herpesviridae; Paramyxoviridae; Poxviridae*
Strigiformes	Strigidae	Owls	225	*Flaviviridae*
Suliformes	Sulidae	Cormorants	10	*Flaviviridae*

1. Clements, J.; Schulenberg, T.; Iliff, M.; Billerman, S.; Fredericks, T.; Gerbracht, J.; Woods, C. Checklist of Birds of the World Available online: https://www.birds.cornell.edu/clementschecklist/download/. (accessed on 19 July 2023).
2. Williams, R.A.J.; Benitez, L. Chapter 5: Avian Poxvirus. In *Ecology of Wild Bird Diseases*; S. Fereidouni, Ed.,CRC Press: Boca Raton, USA, In press; pp. 154–176 ISBN 978-0-8153-7945-4.
