# Supplementary material for: Emerging and Novel Viruses in Passerine Birds
Source: Microorganisms. 2023 Sep 20;11(9):2355. doi: 10.3390/microorganisms11092355 (PMC10536639; doi:10.3390/microorganisms11092355)
Supplement: Supplementary file 1 [file microorganisms-11-02355-s001.zip › Supplementary_Material_TableS3.pdf]

EMERGING AND NOVEL VIRUSES IN PASSERINE BIRDS

Richard AJ Williams[1,3]; Christian Sánchez[1], Ana Doménech [2,3], Ricardo Madrid[1,3]; Sergio Fandiño[2,3]; Pablo Cea-Calleja[1,3];, Esperanza Gomez-Lucia[2,3], Laura Benítez[1,3]

[1]*Department of Genetics, Physiology, and Microbiology, School of Biology, Complutense University of Madrid (UCM), C. de José Antonio Nováis, 12, 28040, Madrid, Spain*

[2]*Deparment of Animal Health, Veterinary Faculty, Complutense University of Madrid, Av. Puerta de Hierro, s/n, 28040, Madrid, Spain*

[3] *"Animal viruses" Research Group, Complutense University of Madrid, Madrid, Spain*

SUPPLEMENTARY MATERIAL: TABLES S3a AND S3b: West Nile Virus (WNV) detection records in class Aves

SUPPLEMENTARY TABLE S3a: WNV detection records recovered from class Aves, using PCR techniques or serology (mainly neutralization, but also cELISA). WNV, or antibodies to it, has been recovered from at least 392 bird species, from 67 families and 27 orders. This list is a minimum figure and is likely to be very much higher. WNV has been detected in 156 passerines (39.8% of WNV positive bird species), which is low considering passerines make up about 60% of bird species.

Order	Family	Species	Common name	Reference
Accipitriformes	Accipitridae	*Accipiter cooperii*	Cooper's hawk	1
		Accipiter gentilis	Northern Goshawk	1
		Accipiter nisus	Sparrowhawk	2
		Accipiter striatus	Sharp-shinned Hawk	1

		Aegypius monachus	Cinereous Vulture	1
		Aquila adalberti	Spanish imperial eagle	3
		Aquila audax	Wedge-tailed Eagle	1
		Aquila chrysaetos	Golden eagle	1
		Aquila nippalensis	Steppe eagle	1
		Buteo buteo	Buzzard	4
		Buteo jamaicensis	Red-tailed Hawk	1
		Buteo lagopus	Rough-legged Hawk	1
		Buteo lineatus	Red-shouldered Hawk	1
		Buteo platypterus	Broad-winged Hawk	1
		Buteo regalis	Ferruginous Hawk	1
		Buteo swainsoni	Swainson's Hawk	1
		Buteogallus anthracinus	Common Black-Hawk	1
		Circaetus gallicus	Short-toed snake Eagle	2
		Circus aeruginosus	Marsh harrier	2
		Circus cyaneus	Hen harrier	5
		Circus hudsonius	Northern Harrier	1
		Circus pygargus	Montagu's harrier	4
		Elanoides forficatus	Swallow-tailed Kite	1
		Elanus leucurus	White-tailed Kite	1
		Gypaetus barbatus	Bearded vulture	6
		Gyps fulvus	Griffon vulture	2
		Haliaeetus leucocephalus	Bald Eagle	1
		Hieraaetus fasciatus	Bonelli's eagle	4
		Hieraaetus pennatus	Booted Eagle	7
		Ictinia mississippiensis	Mississippi Kite	1
		Milvus migrans	Black kite	2
		Milvus milvus	Red kite	2
		Neophron percnopterus	Egyptian vulture	4

		Parabuteo unicinctus	Harris' Hawk	1
		Pernis apivorus	Honey buzzard	8
	Cathartidae	*Cathartes aura*	Turkey Vulture	1
		Coragyps atratus	Black Vulture	1
		Gymnogyps californianus	California Condor	1
	Pandionidae	*Pandion haliaetus*	Osprey	1
Anseriformes	Anatidae	*Aix sponsa*	Wood Duck	1
		Anas acuta	Northern Pintail	1
		Anas carolinensis	Green-winged Teal	1
		Anas fulvigula	Mottled Duck	1
		Anas penelope	Eurasian Wigeon	1
		Anas platyrhynchos	Mallard	1
		Anas undulata	Yellow-billed Duck	1
		Anser albifrons	Greater White-fronted Goose	1
		Anser anser	Greylag geese	9
		Anser caerulescens	Snow Goose	1
		Anser canagicus	Emperor Goose	1
		Anser cygnoides domesticus	Chinese Goose	1
		Aythya affinis	Lesser Scaup	1
		Aythya marila	Greater Scaup	1
		Aythya valisineria	Canvasback	1
		Branta canadensis	Canada Goose	1
		Branta hutchinsii	Cackling Goose	1
		Branta ruficollis	Red-breasted Goose	1
		Branta sandvicensis	Hawaiian Goose	1
		Bucephala albeola	Bufflehead	1
		Bucephala clangula	Common Goldeneye	1
		Cairina moschata	Muscovy Duck	1
		Chen canagica	Emperor Goose	10

		Cygnus columbianus	Tundra Swan	1
		Cygnus olor	Mute Swan	1
		Lophodytes cucullatus	Hooded Merganser	1
		Marmoronetta angustirostris	Marbled duck	7
		Mergellus albellus	Smew	1
		Mergus merganser	Common Merganser	1
		Oxyura jamaicensis	Ruddy Duck	1
		Spatula cyanoptera	Cinnamon Teal	1
		Spatula puna	Puna Teal	1
		Speculanas specularis	Bronze-winged Duck	1
Apodiformes	Trochilidae	*Archilochus alexandri*	Black-chinned Hummingbird	1
		Archilochus colubris	Ruby-throated Hummingbird	1
		Calypte anna	Anna's Hummingbird	1
		Calypte costae	Costa's Hummingbird	1
		Chaetura pelagica	Chimney Swift	1
		Selasphorus rufus	Rufous Hummingbird	1
	Bucoridae	*Bucorvus abyssinicus*	Abyssinian Ground-Hornbill	1
Bucerotiformes	Upupidae	*Upupa epops*	Hoopoe	11
Caprimulgiformes	Caprimulgidae	*Antrostomus vociferus*	Whip-poor-will	1
		Caprimulgus ruficollis	Red-necked Nightjar	5
		Chordeiles acutipennis	Lesser Nighthawk	1
		Chordeiles minor	Common Nighthawk	1
Casuariiformes	Casuariidae	*Dromaius novaehollandiae*	Emu	1
Charadriiformes	Alcidae	*Uria aalge*	Common Murre	1
	Aramidae	*Aramus guarauna*	Limpkin	1
	Charadriidae	*Charadrius melodus*	Piping Plover	1
		Charadrius vociferus	Killdeer	1
	Laridae	*Hydroprogne caspia*	Caspian Tern	1
		Larosterna inca	Inca Tern	1

		Larus argentatus	Herring Gull	1
		Larus atricilla	Laughing Gull	9
		Larus californicus	California Gull	1
		Larus delawarensis	Ring-billed Gull	1
		Larus glaucescens	Glaucous-winged Gull	1
		Larus glaucoides thayeri	Thayer's Gull	1
		Larus marinus	Great Black-backed Gull	1
		Larus michahellis	Yellow-legged gull	12
		Leucophaeus atricilla	Laughing Gull	1
		Rynchops niger	Black Skimmer	1
		Sternula antillarum	Least Tern	1
	Scolopacidae	*Arenaria interpres*	Ruddy Turnstone	1
		Calidris mauri	Western Sandpiper	1
Ciconiformes	Ciconiidae	*Ciconia ciconia*	White stork	7
	Ciconiidae	*Ciconia nigra*	Black stork	2
Columbiformes	Columbidae	*Columba livia*	Rock Pigeon	1
		Columba palumbus	Wood pigeon	8
		Columbina inca	Inca Dove	1
		Columbina passerina	Common Ground-Dove	1
		Patagioenas fasciata	Band-tailed Pigeon	1
		Patagioenas leucocephala	White-crowned Pigeon	1
		Streptopelia decaocto	Eurasian Collared-Dove	1
		Streptopelia risoria	Ringed Turtle-Dove	1
		Streptopelia turtur	Turtle dove	9
		Zenaida asiatica	White-winged Dove	1
		Zenaida aurita	Zenaida Dove	1
		Zenaida macroura	Mourning Dove	1
Coraciiformes	Alcedinidae	*Alcedo atthis*	Common kingfisher	9
		Megaceryle alcyon	Belted Kingfisher	1

		Todiramphus sp.	Micronesian Kingfisher	1
Cuculiformes	Cuculidae	*Coccyzus americanus*	Yellow-billed Cuckoo	1
		Geococcyx californianus	Greater Roadrunner	1
Falconiformes	Falconidae	*Falco columbarius*	Merlin	1
		Falco eleonorae	Eleanora's falcon	13
		Falco mexicanus	Prairie Falcon	1
		Falco naumanni	Lesser kestrel	7
		Falco peregrinus	Peregrine Falcon	1
		Falco rusticolus	Gyrfalcon	1
		Falco sparverius	American Kestrel	1
		Falco subbuteo	Eurasian hobby	5
		Falco tinnunculus	Kestrel	7
Galliformes	Odontophoridae	*Callipepla californica*	California Quail	1
		Colinus virginianus	Northern Bobwhite	1
		Oreortyx pictus	Mountain Quail	1
	Phasianidae	*Alectoris chukar*	Chukar	1
		Alectoris rufa	Red-legged partridge	14
		Bonasa umbellus	Ruffed Grouse	1
		Centrocercus urophasianus	Greater Sage-Grouse	1
		Crossoptilon auritum	Blue-eared Pheasant	1
		Gallus gallus domesticus	Domestic Chicken	1
		Lophophorus impejanus	Impeyan Pheasant	1
		Lophophorus sp.	Monal Pheasant	1
		Meleagris gallopavo	Wild Turkey	1
		Pavo cristatus	Common Peafowl	1
		Phasianus colchicus	Ring-necked Pheasant	1
		Tragopan blythii	Blythe's Tragopan	1
		Tragopan satyra	Satyr Tragopan	1
		Tympanuchus cupido	Greater Prairie-Chicken	1

Gaviiformes	Gaviidae	*Gavia immer*	Common Loon	1
Gruiformes	Gruidae	*Antigone canadensis*	Sandhill Crane	1
		Grus americana	Whooping Crane	1
		Grus canadensis	Sanhill Crane	9
	Rallidae	*Fulica americana*	American Coot	1
		Fulica atra	Common coot	15
		Gallinula chloropus	Common Moorhen	1
		Porphyrio martinica	Purple Gallinule	1
		Porzana carolina	Sora	1
		Rallus crepitans	Clapper Rail	1
		Rallus limicola	Virginia Rail	1
Otidiformes	Otididae	*Otis tarda*	Great bustard	2
Passeriformes	Acrocepahlidae	*Acrocephalus scirpaceus*	Eurasian reed warbler	11
	Aegithalidae	*Psaltriparus minimus*	Bushtit	1
	Bombycillidae	*Bombycilla cedrorum*	Cedar Waxwing	1
	Cardinalidae	*Cardinalis cardinalis*	Northern Cardinal	1
		Passerina amoena	Lazuli Bunting	1
		Pheucticus ludovicianus	Rose-breasted Grosbeak	1
		Pheucticus melanocephalus	Black-headed Grosbeak	1
		Piranga ludoviciana	Western Tanager	1
		Piranga olivacea	Scarlet Tanager	1
		Spiza americana	Dickcissel	1
		Thraupis palmarum	Palm Tanager	1
	Cettidae	*Cettia cetti*	Cetti's warbler	16
	Cinclidae	*Cinclus mexicanus*	American Dipper	1
	Corvidae	*Aphelocoma californica*	Western Scrub-Jay	1
		Aphelocoma wollweberi	Mexican Jay	1
		Corvus brachyrhynchos	American Crow	1
		Corvus corax	Common Raven	1

		Corvus cornix	Hooded Crow	1
		Corvus cryptoleucus	Chihuahuan Raven	1
		Corvus frugilegus	Rook	9
		Corvus ossifragus	Fish Crow	1
		Cyanocitta cristata	Blue Jay	1
		Cyanocitta stelleri	Steller's Jay	1
		Cyanopica cyanus	Azure-winged magpie	11
		Garrulus glandarius	Eurasian Jay	1
		Gymnorhinus cyanocephalus	Pinyon Jay	1
		Nucifraga columbiana	Clark's Nutcracker	1
		Pica hudsonia	Black-billed Magpie	1
		Pica nuttalli	Yellow-billed Magpie	1
		Pica pica	Black-billed magpie	1
	Estrildidae	*Amandava amandava*	Red avadavat	16
		Chloebia gouldiae	Gouldian Finch	1
		Estrilda astrild	Common waxbill	16
		Lonchura punctulata	Nutmeg Mannikin	1
		Lonchura striata domestica	Society Finch	1
		Taeniopygia sp	Zebra Finch	1
	Fringillidae	*Carduelis carduelis*	European Goldfinch	1
		Haemorhous cassinii	Cassin's Finch	1
		Haemorhous mexicanus	House Finch	1
		Haemorhous purpureus	Purple Finch	1
		Hesperiphona vespertina	Evening Grosbeak	1
		Loxia curvirostra	Red Crossbill	1
		Serinus canaria forma *domestica*	Common Canary	1
		Spinus pinus	Pine Siskin	1
		Spinus psaltria	Lesser Goldfinch	1
		Spinus tristis	American Goldfinch	1

	Hirundidae	*Delichon urbicum*	House martin	9
		Hirundo rustica	Barn Swallow	1
		Petrochelidon pyrrhonota	Cliff Swallow	1
		Progne subis	Purple Martin	1
		Riparia riparia	Sand martin	1
		Tachycineta bicolor	Tree Swallow	1
	Icteridae	*Agelaius phoeniceus*	Red-winged Blackbird	1
		Agelaius tricolor	Tricolored Blackbird	1
		Dolichonyx oryzivorus	Bobolink	1
		Euphagus carolinus	Rusty Blackbird	1
		Euphagus cyanocephalus	Brewer's Blackbird	1
		Icterus bullockii	Bullock's Oriole	1
		Icterus cucullatus	Hooded Oriole	1
		Icterus galbula	Baltimore Oriole	1
		Icterus spurius	Orchard Oriole	1
		Molothrus aeneus	Bronzed Cowbird	1
		Molothrus ater	Brown-headed Cowbird	1
		Quiscalus major	Boat-tailed Grackle	1
		Quiscalus mexicanus	Great-tailed Grackle	1
		Quiscalus quiscula	Common Grackle	1
		Sturnella neglecta	Western Meadowlark	1
	Lanidae	*Lanius ludovicianus*	Loggerhead Shrike	1
		Lanius senator	Woodchat shrike	17
	Mimidae	*Dumetella carolinensis*	Gray Catbird	1
		Mimus polyglottos	Northern Mockingbird	1
		Toxostoma lecontei	LeConte's Thrasher	1
		Toxostoma rufum	Brown Thrasher	1
	Motacillidae	*Motacilla flava*	Western yellow wagtail	11
	Muscicapidae	*Erithracus rubecula*	European robin	9

		Phoenicurus ochruros	Black redstart	17
		Phoenicurus phoenicurus	Common redstart	17
	Paridae	*Baeolophus bicolor*	Tufted Titmouse	1
		Baeolophus inornatus	Oak Titmouse	1
		Poecile atricapillus	Black-capped Chickadee	1
		Poecile carolinensis	Carolina Chickadee	1
		Poecile gambeli	Mountain Chickadee	1
		Poecile rufescens	Chestnut-backed Chickadee	1
		Sittiparus varius	Varied Tit	1
	Parulidae	*Cardellina canadensis*	Canada Warbler	1
		Cardellina pusilla	Wilson's Warbler	1
		Geothlypis formosa	Kentucky Warbler	1
		Geothlypis tolmiei	MacGillivray's Warbler	1
		Geothlypis trichas	Common Yellowthroat	1
		Leiothlypis celata	Orange-crowned Warbler	1
		Leiothlypis peregrina	Tennessee Warbler	1
		Leiothlypis ruficapilla	Nashville Warbler	1
		Parkesia noveboracensis	Northern Waterthrush	1
		Seiurus aurocapilla	Ovenbird	1
		Setophaga americana	Northern Parula	1
		Setophaga caerulescens	Black-throated Blue Warbler	1
		Setophaga citrina	Hooded Warbler	1
		Setophaga coronata	Yellow-rumped Warbler	1
		Setophaga dominica	Yellow-throated Warbler	1
		Setophaga nigrescens	Black-throated Gray Warbler	1
		Setophaga petechia	Yellow Warbler	1
		Setophaga striata	Blackpoll Warbler	1
		Setophaga townsendi	Townsend's Warbler	1
	Passerellidae	*Chondestes grammacus*	Lark Sparrow	1

		Junco hyemalis	Dark-eyed Junco	1
		Melospiza georgiana	Swamp Sparrow	1
		Melospiza lincolnii	Lincoln's Sparrow	1
		Melospiza melodia	Song Sparrow	1
		Melozone crissalis	California Towhee	1
		Passerculus sandwichensis	Savannah Sparrow	1
		Passerella iliaca	Fox Sparrow	1
		Pipilo erythrophthalmus	Eastern Towhee	1
		Pipilo maculatus	Spotted Towhee	1
		Spizella atrogularis	Black-chinned Sparrow	1
		Spizella passerina	Chipping Sparrow	1
		Spizella pusilla	Field Sparrow	1
		Zonotrichia atricapilla	Golden-crowned Sparrow	1
		Zonotrichia leucophrys	White-crowned Sparrow	1
	Passeridae	*Passer domesticus*	House Sparrow	1
	Ploceidae	*Euplectes afer*	Yellow-crowned bishop	16
	Sittidae	*Sitta canadensis*	Red-breasted Nuthatch	1
		Sitta carolinensis	White-breasted Nuthatch	1
		Sitta pygmaea	Pygmy Nuthatch	1
	Sturnidae	*Sturnus vulgaris*	European Starling	1
	Sylviidae	*Sylvia borin*	Garden warbler	17
		Sylvia melanocephala	Sardinian warbler	16
	Troglodytidae	*Campylorhynchus brunneicapillus*	Cactus Wren	1
		Salpinctes obsoletus	Rock Wren	1
		Thryothorus ludovicianus	Carolina Wren	1
		Troglodytes aedon	House Wren	1
		Troglodytes hiemalis	Winter Wren	1
	Turdidae	*Catharus fuscescens*	Veery	1
		Catharus guttatus	Hermit Thrush	1

		Catharus minimus	Gray-cheeked Thrush	1
		Catharus ustulatus	Swainson's Thrush	1
		Hylocichla mustelina	Wood Thrush	1
		Ixoreus naevius	Varied Thrush	1
		Sialia currucoides	Mountain Bluebird	1
		Sialia mexicana	Western Bluebird	1
		Sialia sialis	Eastern Bluebird	1
		Turdus merula	Blackbird	11
		Turdus migratorius	American Robin	1
	Tyrannidae	*Contopus cooperi*	Olive-sided Flycatcher	1
		Empidonax difficilis	Pacific-slope Flycatcher	1
		Empidonax hammondii	Hammond's Flycatcher	1
		Empidonax traillii	Willow Flycatcher	1
		Myiarchus cinerascens	Ash-throated Flycatcher	1
		Myiarchus crinitus	Great Crested Flycatcher	1
		Sayornis nigricans	Black Phoebe	1
		Sayornis phoebe	Eastern Phoebe	1
		Tyrannus forficatus	Scissor-tailed Flycatcher	1
		Tyrannus tyrannus	Eastern Kingbird	1
		Tyrannus verticalis	Western Kingbird	1
	Vireonidae	*Vireo altiloquus*	Black-whiskered Vireo	1
		Vireo gilvus	Warbling Vireo	1
		Vireo olivace	Red-eyed Vireo	1
Pelecaniformes	Ardeidae	*Ardea alba*	Great Egret	1
		Ardea heodias	Great Blue Heron	1
		Bubulcus ibis	Western cattle egret	1
		Butorides virescens	Green Heron	1
		Egretta garzetta	Little egret	9
		Egretta thula	Snowy Egret	1

		Ixobrychus exilis	Least Bittern	1
		Nyctanassa violacea	Yellow-crowned Night-Heron	1
		Nycticorax nycticorax	Black-crowned Night Heron	1
	Pelecanidae	*Pelecanus erythrorhynchos*	American White Pelican	1
	Threskiornithidae	***Eudocimus ruber***	Scarlet Ibis	1
		Platalea leucorodia	Spoonbill	4
		Plegadis chihi	White-faced Ibis	1
		Plegadis falcinellus	Glossy ibis	12
Phoenicopteriformes	Phoenicopteridae	*Phoenicopterus chilensis*	Chilean Flamingo	1
		Phoenicopterus roseus	Greater flamingo	1
Piciformes	Picidae	*Colaptes auratus*	Northern Flicker	1
		Dryobates nuttallii	Nuttall's Woodpecker	1
		Dryobates pubescens	Downy Woodpecker	1
		Dryobates villosus	Hairy Woodpecker	1
		Melanerpes carolinus	Red-bellied Woodpecker	1
		Melanerpes erythrocephalus	Red-headed Woodpecker	1
		Melanerpes formicivorus	Acorn Woodpecker	1
		Melanerpes lewis	Lewis' Woodpecker	1
		Melanerpes uropygialis	Gila Woodpecker	1
		Sphyrapicus ruber	Red-breasted Sapsucker	1
		Sphyrapicus varius	Yellow-bellied Sapsucker	1
Podicipediformes	Podicipedidae	*Aechmophorus clarkii*	Clark's Grebe	1
		Podilymbus podiceps	Pied-billed Grebe	1
Psittaciformes	Cacatuidae	*Cacatua sp.*	Cockatoo	1
	Psittacidae	*Amazona viridigenalis*	Red-crowned Parrot	1
		Brotogeris sp.	Canary-winged Parakeet	1
		Forpus coelestis	Pacific Parrotlet	1
		Psittacara finschi	Crimson-fronted Parakeet	1
		Psittacus erithacus	African Grey Parrot	1

		Rhynchopsitta pachyrhyncha	Thick-billed Parrot	1
		Thectocercus acuticaudatus	Blue-crowned Conure	1
	Psittaculidae	*Coracopsis vasa*	Greater vasa parrot	9
		Eos bornea	Red Lory	1
		Eos reticulata	Blue-streaked Lory	1
		Eos squamata	Violet-necked Lorikeet	1
		Lorius lory	Black-capped Lory	1
		Melopsittacus undulatus	Budgerigar	1
		Nymphicus hollandicus	Cockatiel	1
		Platycercus adscitus	Pale-headed Rosella	1
		Platycercus elegans	Crimson Rosella	1
		Pseudeos fuscata	Dusky Lory	1
		Trichoglossus moluccanus	Rainbow Lorikeet	1
Sphenisciformes	Spheniscidae	*Spheniscus demersus*	African Penguin	1
		Spheniscus humboldti	Humboldt Penguin	1
Strigiformes	Strigidae	*Aegolius acadicus*	Northern Saw-whet Owl	1
		Aegolius funereus	Boreal Owl	1
		Asio flammeus	Short-eared Owl	1
		Asio otus	Long-eared Owl	1
		Athene cunicularia	Burrowing Owl	1
		Bubo bubo	Eagle owl	2
		Bubo scandiacus	Snowy Owl	1
		Bubo virginianus	Great Horned Owl	1
		Megascops asio	Eastern Screech-Owl	1
		Megascops kennicottii	Western Screech-Owl	1
		Micrathene whitneyi	Elf Owl	1
		Otus scops	Eurasian scops owl	18
		Psiloscops flammeolus	Flammulated Owl	1
		Strix aluco	Tawny Owl	1

		Strix nebulosa	Great Gray Owl	1
		Strix occidentalis	Spotted Owl	1
		Strix varia	Barred Owl	1
		Surnia ulula	Northern Hawk-Owl	1
	Tytonidae	*Tyto alba*	Barn Owl	1
Struthiorniformes	Struthionidae	*Struthio camelus*	Ostrich	10
Suliformes	Phalacrocoracidae	*Leucocarbo bougainvillii*	Guanay Cormorant	1
		Nannopterum auritum	Double-crested Cormorant	1
		Phalacrocorax carbo	Great cormorant	9
		Urile pelagicus	Pelagic Cormorant	1
Tinamiformes	Tinamidae	*Eudromia elegans*	Elegant Crested Tinamou	1

SUPPLEMENTARY TABLE S3b: summary of WNV detections in avian orders showing the number of positive families and species in each order, and the percent of total detections for the order.

**Order**	**# of WNV positive families**	**# of WNV positive species**	**% of total WNV detections**
Accipitriformes	3	39	9.9
Anseriformes	1	33	8.4
Apodiformes	1	6	1.5
Bucerotiformes	2	2	0.5
Caprimulgiformes	1	4	1.0
Casuariiformes	1	1	0.3
Charadriiformes	5	19	4.8
Ciconiformes	1	2	0.5
Columbiformes	1	12	3.1
Coraciiformes	1	3	0.8
Cuculiformes	1	2	0.5

Falconiformes	1	9	2.3
Galliformes	2	17	4.3
Gaviiformes	1	1	0.3
Gruiformes	2	10	2.6
Otidiformes	1	1	0.3
Passeriformes	27	156	39.8
Pelecaniformes	3	14	3.6
Phoenicopteriformes	1	2	0.5
Piciformes	1	11	2.8
Podicipediformes	1	2	0.5
Psittaciformes	3	19	4.8
Sphenisciformes	1	2	0.5
Strigiformes	2	19	4.8
Struthiorniformes	1	1	0.3
Suliformes	1	4	1.0
Tinamiformes	1	1	0.3

References

1. Centers for Disease Control and Prevention. 2016. Species of dead birds in which West Nile virus has been detected, United States, 1999–2016. https://www.cdc.gov/westnile/resources/pdfs/Bird%20Species%201999-2012.pdf. Accessed June 2023
2. Bravo-Barriga, D., et al., *West Nile and Usutu virus infections in wild birds admitted to rehabilitation centres in Extremadura, western Spain, 2017-2019.* Veterinary Microbiology, 2021. **255**: p. 10.
3. Höfle, U., et al., *West Nile virus in the endangered Spanish imperial eagle.* Veterinary Microbiology, 2008. **129**(1-2): p. 171-178.
4. Garcia-Bocanegra, I., et al., *High exposure of West Nile virus in equid and wild bird populations in Spain following the epidemic outbreak in 2020.* Transboundary and Emerging Diseases, 2022. **69**(6): p. 3624-3636.
5. Alba, A., et al., *Ecological surveillance for West Nile in Catalonia (Spain), learning from a five-year period of follow-up.* Zoonoses and Public Health, 2014. **61**(3): p. 181-191.

6. Napp, S., et al., *Widespread circulation of Flaviviruses in horses and birds in northeastern Spain (Catalonia) between 2010 and 2019.* Viruses-Basel, 2021. **13**(12): p. 13.
7. Lopez, G., et al., *Prevalence of West Nile virus neutralizing antibodies in Spain is related to the behavior of migratory birds.* Vector-Borne and Zoonotic Diseases, 2008. **8**(5): p. 615-621
8. Ziegler, U., et al., *Spread of West Nile virus and Usutu virus in the German bird population, 2019-2020.* Microorganisms, 2022. **10**(4): p. 21.
9. CABI (2022) 'West Nile virus', CABI Compendium. CABI International. doi: 10.1079/cabicompendium.59558.
10. Cano-Terriza, D., et al., *Epidemiological survey of zoonotic pathogens in feral pigeons (Columba livia var. domestica) and sympatric zoo species in Southern Spain.* Comparative Immunology Microbiology and Infectious Diseases, 2015. **43**: p. 22-27.
11. Ferraguti, M., et al., *West Nile virus-neutralizing antibodies in wild birds from southern Spain.* Epidemiology and Infection, 2016. **144**(9): p. 1907-1911.
12. Figuerola, J., et al., *Prevalence of West Nile virus neutralizing antibodies in colonial aquatic birds in southern Spain.* Avian Pathology, 2007. **36**(3): p. 209-212.
13. Gangoso, L., et al., *Prevalence of neutralizing antibodies to West Nile virus in Eleonora's Falcons in the Canary Islands.* Journal of Wildlife Diseases, 2010. **46**(4): p. 1321-1324.
14. Llorente, F., et al., *Flaviviruses in game birds, southern Spain, 2011-2012.* Emerging Infectious Diseases, 2013. **19**(6): p. 1023-5.
15. Figuerola, J., et al., *Seroconversion in wild birds and local circulation of West Nile virus, Spain.* Emerging Infectious Diseases, 2007. **13**(12): p. 1915-1917.
16. Marzal, A., et al., *Circulation of zoonotic flaviviruses in wild passerine birds in Western Spain.* Veterinary Microbiology, 2022. **268**: p. 5.
17. Lopez, G., et al., *Incidence of West Nile virus in birds arriving in wildlife rehabilitation centers in southern Spain.* Vector-Borne and Zoonotic Diseases, 2011. **11**(3): p. 285-290.
18. Jurado-Tarifa, E., et al., *Monitoring of West Nile virus, Usutu virus and Meaban virus in waterfowl used as decoys and wild raptors in southern Spain.* Comparative Immunology Microbiology and Infectious Diseases, 2016. **49**: p. 58-64.
