# Supplementary material for: Emerging and Novel Viruses in Passerine Birds
Source: Microorganisms. 2023 Sep 20;11(9):2355. doi: 10.3390/microorganisms11092355 (PMC10536639; doi:10.3390/microorganisms11092355)
Supplement: Supplementary file 1 [file microorganisms-11-02355-s001.zip › Supplementary_Material_TableS4.pdf]

EMERGING AND NOVEL VIRUSES IN PASSERINE BIRDS

Richard AJ Williams[1,3]; Christian Sánchez[1], Ana Doménech [2,3], Ricardo Madrid[1,3]; Sergio Fandiño[2,3]; Pablo Cea-Calleja[1,3]; Esperanza Gomez-Lucia[2,3], Laura Benítez[1,3]

[1]*Department of Genetics, Physiology, and Microbiology, School of Biology, Complutense University of Madrid (UCM), C. de José Antonio Nováis, 12, 28040, Madrid, Spain*

[2]*Deparment of Animal Health, Veterinary Faculty, Complutense University of Madrid, Av. Puerta de Hierro, s/n, 28040, Madrid, Spain*

[3] *"Animal viruses" Research Group, Complutense University of Madrid, Madrid, Spain*

SUPPLEMENTARY MATERIAL: TABLES S4a AND S4b: Usutu virus (USUV) detection records in class Aves

SUPPLEMENTARY TABLE S4a: USUV detection records recovered from class Aves, using PCR techniques or neutralization serology. USUV, or antibodies to it, has been recovered from at least 100 bird species, from 34 families and 18 orders. This list is a minimum figure, based on just 15 articles: a start. Just under half the detections (46%) are from order Passeriformes. USUV has only been detected from one study for the majority of species. It is thus striking that USUV has been detected in common blackbird in 11 of the 15 studies we used to compile this list. The column "Status" records whether the individual was captive, invasive or wild, and is left blank when the status is not clear, or with "Captive?" for exotic species, where the authors were not explicit.

Order	Family	Species	Status	Region	Reference
Accipitriformes	Accipitridae	*Accipiter gentilis*		Europe: Germany	7
		Aquila nipalensis		Europe: Germany	7
		Buteo buteo		Europe: Germany	7
		Circus aeruginosus	Wild	Europe: Austria	9
		Gypaetus barbatus	Wild	Europe: Austria	9
		Neophron percnopterus	Captive	Europe: Austria	3
		Parabuteo unicinctus		Europe: Germany	7
		Pernis apivorus		Europe: Germany	7
Anseriformes	Anatidae	*Alopochen aegyptiaca*		Europe: Belgium	2
		Anas platyrhynchos	Wild	Europe: Belgium	2
		Anser anser		Europe: Germany	7
		Branta canadensis	Wild	Europe: Belgium	2
		Branta ruficollis	Captive	Europe: Switzerland	3
		Cygnus olor	Wild	Europe: Belgium	2
		Dendrocygna viduata	Wild	Africa: Madagascar	6
		Mergus squamatus		Europe: Germany	7
		Spatula hottentota	Wild	Africa: Madagascar	6
		Tachyeres pteneres	Captive	Europe: Switzerland	3
		Tadorna ferruginea	Captive	Europe: Switzerland	3
Apodiformes	Apodidae	*Apus apus*	Wild	Europe: Belgium	2
Bucerotiformes	Bucerotidae	*Bycanistes sharpii*	Wild	Africa: Nigeria	11
Ciconiiformes	Ciconiidae	*Ciconia ciconia*	Captive	Europe: Austria	3
		Leptoptilos crumenifer	Captive	Europe: Switzerland	3
Columbiformes	Columbidae	*Columba livia domestica*		Europe: Belgium	2
		Columba palumbus		Europe: Germany	7
		Streptopelia decaocto	Wild	Europe: Austria	9
Coraciiformes	Alcedinidae	*Dacelo novaeguineae*	Captive	Europe: Switzerland	3
		Alcedo atthis	Wild	Europe: Germany	1

Falconiformes	Falconidae	*Falco tinnunculus*	Wild	Europe: Austria	9, 10
Galliformes	Phasianidae	*Gallus gallus domesticus*	Captive	Europe: Switzerland	3
		Pavo cristatus		Europe: Austria	9
		Phasianus colchicus		Europe: Austria	9
		Tetrao urogallus		Europe: Germany	7
Passeriformes	Acrocephalidae	*Acrocephalus scirpaceus*	Wild	Europe: Austria	9
	Corvidae	*Coccothraustes coccothraustes*		Europe: Germany	7
		Coloeus monedula	Wild	Europe: Germany	9, 10
		Corvus corone	Wild	Europe: Germany	10
		Corvus corone cornix	Wild	Europe: Austria	9
		Cyanopica cooki	Wild	Europe: Spain	4
		Garrulus glandarius	Wild	Europe: Belgium	2
		Nucifraga caryocatactes		Europe: Germany	7
		Pica pica	Wild	Europe: Belgium	2
		Pyrrhocorax pyrrhocorax		Europe: Germany	7
	Emberizidae	*Emberiza schoeniclus*	Wild	Europe: Austria	9
	Estrildidae	*Amandava amandava*	Invasive	Europe: Spain	4
		Erythrura prasina	Captive	Europe: Germany	12
		Mandingoa nitidula	Captive	Europe: Germany	12
		Taeniopygia guttata	Captive	Europe: Germany	12
	Fringillidae	*Chloris chloris*	Wild	Europe: Switzerland	13
		Chlorophonia occipitalis	Captive	Europe: Germany	12
		Fringilla coelebs	Wild	Europe: Belgium	2
		Pyrrhula erythaca	Captive?	Europe: Germany	10
		Pyrrhula pyrrhula	Wild	Europe: Germany	10
		Serinus canaria domestica	Captive?	Europe: Germany	9, 11
	Hirundidae	*Delichon urbicum*	Wild	Europe: Spain	4, 9
		Hirundo rustica	Wild	Europe: Austria	9
	Laniidae	*Lanius senator*	Wild	Europe: Spain	4

	Motacillidae	*Motacilla alba*	Wild	Europe: Belgium	2
	Muscicapidae	*Erithacus rubecula*	Wild	Europe: Austria	9, 13
		Ficedula hypoleuca	Wild	Europe: Austria	5, 9
		Luscinia megarhynchos	Wild	Europe: Spain	4
		Phoenicurus ochruros	Wild	Europe: Austria	9, 10
	Paridae	*Cyanistes caeruleus*	Wild	Europe: Switzerland	13
		Parus major	Wild	Europe: Austria	5, 9
		Periparus ater		Europe: Germany	7
	Passeridae	*Passer domesticus*	Wild	Europe: Switzerland	10, 13
	Pycnonotidae	*Eurillas virens*	Wild	Africa: Nigeria	11
	Sittidae	*Sitta europaea*	Wild	Europe: Austria	5, 9
	Sturnidae	*Lamprotornis regius*		Europe: Germany	7
		Sturnus vulgaris	Wild	Europe: Austria	9, 10
	Sylviidae	*Sylvia atricapilla*	Wild	Europe: Austria	4, 9
		Sylvia borin	Wild	Europe: Austria	9
		Sylvia communis	Wild	Europe: Austria	9
		Sylvia curruca	Wild	Europe: Austria	9
	Troglodytidae	*Troglodytes troglodytes*	Wild	Europe: Belgium	1
	Turdidae	*Turdus libonyana*	Wild	Africa: Nigeria	11
		Turdus merula	Wild	Europe: Italy	1, 2, 4, 6, 9, 10, 12, 13, 14, 15
		Turdus philomelos	Wild	Europe: Austria	2, 8, 9, 10
		Turdus pilaris		Europe: Germany	7
Pelecaniformes	Ardeidae	*Ardea cinerea*		Europe: Germany	7
Piciformes	Picidae	*Dendrocopos major*	Wild	Europe: Austria	9, 10
		Picus viridis		Europe: Germany	7
Phoenicopteriformes	Phoenicopteridae	*Phoenicoparrus chilensis*		Europe: Germany	7
		Phoenicopterus ruber	Captive	Europe: Germany	3
Psittaciformes	Cactuidae	*Calyptorhynchus banksii*	Captive	Europe: Germany	12

		Calyptorhynchus baudinii	Captive	Europe: Germany	12
Sphenisciformes	Spheniscidae	*Spheniscus humboldti*	Captive	Europe: Switzerland	3
Strigiformes	Strigidae	*Aegolius funereus*	Captive	Europe: Switzerland	13
		Asio otus	Wild	Europe: Austria	9
		Athene noctua	Wild	Europe: Germany	10
		Bubo bubo	Captive	Europe: Austria	3, 9
		Bubo scandiacus	Captive	Europe: Austria	3
		Glaucidium passerinum	Captive	Europe: Switzerland	2, 13
		Strix aluco	Wild	Europe: Austria	9
		Strix nebulosa	Captive	Europe: Switzerland	10, 13
		Strix uralensis	Captive?	Europe: Austria	3, 9
		Surnia ulula	Captive	Europe: Switzerland	13
	Tytonidae	*Tyto alba*	Wild	Europe: Austria	9

SUPPLEMENTARY TABLE S4b: summary of detections in avian orders showing the number of positive families and species in each order, and the percent of total detections for the order.

**Order**	**# of USUV positive families**	**# of USUV positive species**	**% of total USUV detections**
Accipitriformes	1	8	8
Anseriformes	1	11	11
Apodiformes	1	1	1
Bucerotiformes	1	1	1
Charadriiformes	1	2	2
Ciconiiformes	1	2	2
Columbiformes	1	3	3
Coraciiformes	1	2	2
Falconiformes	1	1	1

Galliformes	1	4	4
Passeriformes	17	46	46
Pelecaniformes	1	1	1
Piciformes	1	2	2
Phoenicopteriformes	1	2	2
Psittaciformes	1	2	2
Sphenisciformes	1	1	1
Strigiformes	2	11	11

References

1. Becker, N., et al., *Epizootic emergence of Usutu virus in wild and captive birds in Germany.* PLoS ONE, 2012. **7**(2): p. e32604.
2. Benzarti, E., et al., *Usutu virus epizootic in Belgium in 2017 and 2018: evidence of virus endemization and ongoing introduction events.* Vector-Borne and Zoonotic Diseases, 2020. **20**(1): p. 43-50.
3. Buchebner, N., et al., *Low Usutu virus seroprevalence in four zoological gardens in central Europe.* BMC Veterinary Research, 2013. **9**: p. 1-7.
4. Bravo-Barriga, D., et al., *Identification of Usutu virus Africa 3 lineage in a survey of mosquitoes and birds from urban areas of western Spain.* Transboundary and Emerging Diseases, 2023. **2023**: p. 10.
5. Chvala, S., et al., *Monitoring of Usutu virus activity and spread by using dead bird surveillance in Austria, 2003–2005.* Veterinary Microbiology, 2007. **122**(3-4): p. 237-245.
6. Chevalier, V., et al., *Serological evidence of West Nile and Usutu viruses circulation in domestic and wild birds in wetlands of Mali and Madagascar in 2008.* International Journal of Environmental Research and Public Health, 2020. **17**(6).
7. Günther, A., et al., *Continuous surveillance of potentially zoonotic avian pathogens detects contemporaneous occurrence of highly pathogenic avian influenza viruses (HPAIV H5) and flaviviruses (USUV, WNV) in several wild and captive birds*. Emerging Microbes & Infections, 2023. ***12***(2): 2231561.
8. Höfle, U., et al., *Usutu virus in migratory song thrushes, Spain.* Emerging Infectious Diseases, 2013. **19**(7): p. 1173-1175.
9. Meister, T., et al., *Serological evidence of continuing high Usutu virus (Flaviviridae) activity and establishment of herd immunity in wild birds in Austria.* Veterinary Microbiology, 2008. **127**(3): p. 237-248.

10. Michel, F., et al., *Evidence for West Nile virus and Usutu virus infections in wild and resident birds in Germany, 2017 and 2018.* Viruses, 2019. **11**(7): p. 674.
11. Nikolay, B., et al., *Usutu Virus in Africa.* Vector-Borne and Zoonotic Diseases, 2011. **11**(11): p. 1417-1423
12. Schmidt, V., et al., *Usutu virus infection in aviary birds during the cold season.* Avian Pathology, 2021. **50**(5): p. 427-435.
13. Steinmetz, H.W., et al., *Emergence and establishment of Usutu virus infection in wild and captive avian species in and around Zurich, Switzerland—genomic and pathologic comparison to other central European outbreaks.* Veterinary Microbiology, 2011. **148**(2-4): p. 207-212.
14. Weissenböck, H., et al., *Usutu virus, Italy, 1996.* Emerging Infectious Diseases, 2013. **19**(2): p. 274.
15. Weissenböck, H., et al., *Emergence of Usutu virus, an African mosquito-borne Flavivirus of the Japanese Encephalitis virus group, Central Europe.* Emerging Infectious Disease journal, 2002. **8**(7): p. 652.
